# Extracts and Gleanings

**Published:** 1873-01

**Authors:** 


### PART II.

### EXTRACTS AND GLEANINGS FROM OUR EXCHANGES.

### *MULTUM IN PARVO.*

*Fever—Treatment of.*

In severe cases of fever, what our efforts should be principally directed to avert is stagnation of the blood in the small vessels, and cessation of the capillary circulation over a considerable part of the body. The principal cause of this is failure of the heart's action. During the critical period, it may be necessary to excite the organ to increased action by the administration of stimulants, and it is very important to watch carefully for any indications of approaching failure.

As bearing upon the important question of restraining extreme heat of the body in disease, we must remember that, whereas the nitrogen-containing tissues are hardly consumed at all in circumstances of health, the febrile state involves a large destruction of them, and this at a time when, from the state of appetite and primary digestion, scarcely any supplies of nitrogenous food can be taken into the system. Every additional degree of fever heat implies so much additional destruction of the most important organs of the body, including the heart and the nerve centres. Excessive heat does not mean plenty of strength, but exactly the opposite; it is an absolute proof that the reserve forces of the body are exceedingly low, and are being constantly and rapidly reduced.

It is a fact which no theory can alter, that, if the temperature of the body rise up to, or above 107°, death is imminent; partly from the effect of the excessive heat on the vitality of organs, and partly from the excessive tissue-change which must be going on to produce this heat. Excessive and very rapid rise of temperature is seen more frequently in acute rheumatism than in any other disease. Dr. Wilson Fox relates a case in which, when the temperature was 107° in the rectum, the patient was placed in a bath at 96°; the heat, however, continuing to rise to 110° in the rectum, iced water was used and ice placed down the spine. In half an hour the temperature was reduced to 103.6°, and in another half hour to 99.5°. During this time six ounces of brandy were given, and subsequently eighteen ounces a day for several days.

The statistics published in Germany, comprising more than a thousand cases of typhoid fever, treated in various hospitals, show that the mortality in that disease has been reduced by more than one-half by the cooling treatment. This consists in not allowing the temperature to rise above a certain standard. If it does, it is at once lowered by baths or other means. This method of treatment is, therefore, not directed against the disease itself, but simply against a symptom, upon which a series of other symptoms depend. The patient is not plunged in a cold bath, but in one ten degrees below the heat of his body. It is only in certain cases that the use of absolutely cold water or ice is required. As many as ten or twelve baths in the twenty-four hours are sometimes required, in order to keep down the excessive fever heat.

There can be no doubt that some cases prove fatal simply from the excessive temperature of the body, consuming the tissues so rapidly. No case can long go on favorably with a temperature of say 106°, and as it is proved by a considerable amount of experience, especially in Germany, that an excessive temperature may, without any danger, be lowered artificially, this plan of treatment is imperatively called for in cases where an excessive temperature exists. Withdrawal of heat must, however, be practiced with judgment, and not too rapidly, as the temperature will then swing back to and even beyond its former place. The rule ought to be, that the withdrawal of heat should be the slowest possible for the due attainment of our end. The patient should be placed in a bath of 100°, and the temperature of this gradually lowered, so that no shock is given to the system. On the removal from the bath, quinine in doses of from five to twenty grains, according to circumstances, as advocated by Binz, will arrest the rapid reproduction of heat. This mode of treatment requires the incessant use of the thermometer, and the personal supervision of the medical attendant. A very interesting and successful illustrative case is related: it was one of cerebral rheumatism, which had supervened upon an ordinary attack of rheumatic fever, without heart affection. The temperature was 105.8°. A bath not being at hand, ice was put in five wide-necked bottles and placed in bed, close to the body, and ice also applied to the head. The cold was thus applied partially, and the same effect produced as by a less degree of cold applied to the whole surface of the body.—*Braithwaite.*

*M. Ollier's Occlusive Dressing.*

In the last two numbers of the Lyon Médical, M. Poncet brings forward some new illustrations of the great advantages attending the employment of M. Alphonse Guérin's mode of dressing wounds with cotton, in association, as recommended by M. Ollier, with the silicate bandage, so as to constitute what the latter surgeon terms *occlusion immobile.* The object of these papers is, however, not to set forth the general advantages of this plan, which have now been made pretty widely known, but to show its great utility in warding off those hospital complications which have so often excited terrible effects—pyæmia, erysipelas, and hospital gangrene. During the last half-year, more than one hundred of the *bandages ouatés-silicates* have been applied in M. Ollier's ward (containing one hundred patients) to every wounded surface, and not one of these patients has succumbed to pyæmia, or been attacked by erysipelas. The bandage in these cases has been kept on for a week or a fortnight, and sometimes for three or more weeks; all accidents from such retention being guarded against by paying attention to the pulse, temperature and feelings of the patient. The bandage has had to be removed wholly or in part in a few cases, on account of the imperfect way in which it had been applied. In all cases, wherever possible, such removal has not been performed in the wards where the other patients were; the wound being also kept exposed for the shortest possible time; and the quite new, fine cotton taken only from the parcel as wanted. The portion of this brought in immediate contact with the wound has usually been impregnated with carbolic acid and alcohol. In this mode of dressing, the minutest details must be considered, and the great object of exclusion of air can only be attained by the super-position of several thick layers of cotton, which must reach very far beyond the surface of the wound. Thus, after an amputation of the leg, it does not suffice to cover the stump with the silicated bandage, but this must also comprise the thigh and the trunk. Without such precautions, it becomes a bad mode of dressing, retaining pus which has undergone change from action of the air in contact with the wound. Before the bandage is applied, as many arteries as possible should be secured, and for this purpose the wound should be left exposed for fifteen to twenty minutes.

M. Poncet gives various details concerning the chief classes of wounds which were treated during this period; and he observes that if these cases were taken as conclusive, this procedure might be pronounced an absolute preservative against these complications. No case of pyæmia, erysipelas or hospital gangrene was observed in cases so protected, although the last two affections prevailed in the ward. However, he wishes no exaggeration upon the subject, and observes that, while failure will occur wherever the bandage is imperfectly applied, even when its adaptation is perfect, we only place the wound in the very best condition for healing by preventing complications from without. Its healing, however, of course, depends also upon other circumstances; it is a means especially adapted for hospital practice, and is not required in cases treated in localities where the air is not likely to be contaminated. The cases M. Poncet relates were also treated in winter, but in summer the dressing may have to be removed every five or six instead of every fifteen or twenty days.—*Medical Times and Gazette, Aug.* 3, 1872.

*Ice to the Neck in Hysteria, and in Suspended Respiration.*

J. T. Rothrock, M.D., (Philadelphia Medical Times, November 2), relates the following cases: In March, 1872, I was requested by a dental friend to administer an anæsthetic to a patient, for the purpose of extracting a number of teeth. Notwithstanding the dread of the patient in regard to inhalation of chloroform or ether, caused by supposed cardiac and pulmonary trouble, (a dread in which I did not share) I discovered on actual trial that she evinced a remarkable toleration of it, so much so that an unusually large quantity was required to produce complete anæsthesia. The teeth were extracted, and, while we were awaiting a return of consciousness, hysterical symptoms manifested themselves. These culminated in one prolonged, terrific fit of hysterical noisiness. For fully an hour the patient screamed so loudly as to set at rest any lingering doubt as to the healthy condition of her lungs. The usual restoratives were vigorously plied. Antispasmodics in turn failed. Within convenient reach from the window lay some snow. I made a snow ball of it, and on applying it to the forehead obtained some remission in the screams of the patient; but after a faithful trial I found her still unrestored, and still noisy enough to disturb the neighborhood.

I then tried the same remedy to the neck along the line of the sterno-cleido-mastoid muscle. Within a minute she gave a deep sigh, opened her eyes, looked rational, and the hysteria showed evident signs of vanishing, as it did very soon after.

In September of the same year, I was called to see a nervous, delicately-organized woman, who had, on the death of her child, fallen upon the floor in a paroxysm of hysteria. When I reached the house, I found her still lying where she had fallen, insensible, and in a state of clonic spasm, which closely simulated poisoning from strychnia. Mustard was freely applied to the wrists and ankles, and as much bromide of potassium given as could be forced, in concentrated solution, between her firmly-locked jaws. These remedies, on which I have learned to rely in such cases, seemed to fail me utterly here, after a fair, persistent trial. I then applied a lump of ice to either side of the neck, stroking it up and down lightly in the same line as I had the snow-ball in the previous case. Again I obtained a deep, satisfactory sigh within a minute. So long as I allowed her to remain without the ice-application, or applied it to the forehead or face, she ceased to take full inspirations, and went back into spasm. On applying it again, and maintaining its action, the sighs followed, and with them came relaxation of spasm and easy breathing, which within an hour terminated comfortably in consciousness. From what I afterwards learned of this same person, I found that she was in the habit of having these hysterical paroxysms, and also that they never before had terminated so soon as did this one.

Within two weeks of the above case, I administered a mixture of chloroform and ether to a plethoric lady. The anæsthetic was pushed to the point of marked stertorous breathing. Her return to consciousness was so slow as to alarm her friends, and I was urged to "do something." I applied the ice as in the preceding case, and almost instantly the same sigh followed. Keeping up the ice to the neck, she awoke in an unexpectedly short time, considering how profoundly she had been under the influence of the anæsthetic.

These cases are simply three from quite a number I could give. They are sufficient, however, for my purpose.

*A Simple Method of Treating Chronic Diarrhœa.*

A non-professional friend calls our attention to the case of a young lady, twenty-one years of age, in rather delicate health, of frail constitution, and whose mother some years since died in consumption. For two years she has been comparatively an invalid, the result, in a great measure, of chronic diarrhœa. During that time she has been under the care of several physicians, and has taken a variety of medicines, but all without effect. For two months she has been employing a very simple remedy with great relief, viz.: One tablespoonful of arrowroot made into a rather thin gruel with milk, and a tablespoonful of brandy added (or taken separately) morning and evening. Under this treatment she began to improve immediately, and so continued. Her health has returned, and with it color and physical vivacity.—*Boston Medical and Surgical Journal.*

*For Chilblains.*

Oxide of zinc, two parts; tannic acid, one part; glycerine, ten parts; balsam of Peru, eight parts; camphor, four parts. M.—*From l' Union Medicale.*

*Two Cases of Malarial Diarrhœa.*

H. G. Landis, M.D., (Philadelphia Medical Times, November 2, 1872), reports the following cases: Mrs. W. B., æt. 32, was delivered September 10 of her twelfth child, after a hard labor and the use of instruments, due to pelvic deformity and an enormous child. She had repeatedly suffered from similar labors. On the following day she was put on an antiphlogistic course of treatment, in order to be prepared for the inflammation, which seemed, after such a labor, almost inevitable. On the 12th, a small dose of ol. ricin. was taken, after which she had several evacuations. On the 13th, considerable inflammation of the vagina existed, and the bowels became very loose. Hops were applied locally in the form of a poultice, and all other treatment but morphia suspended. The diarrhœa persisting, opium and tannic acid were used freely until the 19th, when both the diarrhœa and vaginitis were at an end. After this she did very well, sitting up on the 24th, and walking out on the 26th, until on the 27th another attack of diarrhœa commenced, occurring every night without either chill or fever, and not troubling her in the daytime; differing in this respect from the former attack, which was irregularly continuous. On October 1, I first became aware of this condition, and gave bismuthi subnitr., myristicæ, aa gr. x., every four hours, which had no effect whatever. Guided only by the periodicity of the attacks, I gave her quinine on the 3d, which promptly stopped the diarrhœa in one day.

D. P., æt. 35, applied for treatment September 21. He had suffered from diarrhœa for three weeks, and was now passing some blood and pus. As in the former case, he was only troubled at night, but, being daily engaged at hard work, was occasionally chilly, and subject to cold perspirations, for which his weakened condition would fully account. He could assign no reason for the attack, and I gave him opium, bismuth and myristicæ, which gave him one night's rest, and then failed to exercise any influence. On the 24th this was changed for quinine in three-grain doses, which immediately stopped the diarrhœa.

*Glycerin Lotion.*

For softening the skin of the face and hands, especially during the commencement of cold weather, and also for allaying the irritation caused by the razor:

Triturate 4½ grains of cochineal with 1½ fluid ounces of boiling water, added gradually; then add 2½ fluid ounces of alcohol. Also make an emulsion of 8 drops of attar of roses with 30 grains of gum Arabic and eight fluid ounces of water; then add 3 fluid ounces of glycerin and 10 fluid drachms of quince mucilage. Mix the two liquids.—*Zeitschrift, etc.,* 1871.

*Syphilitic Ulceration of the Face.*—Clinic of Professor Gross, reported by Dr. Frank Woodbury.

This young married woman has a number of superficial ulcers involving the upper lip and the entire nose, which is partly destroyed. These extend from the eyes down to the mouth, are covered with spoiled lymph, and tell their own story. The patient lives with her husband, who is healthy, and she denies ever having the primary lesion, sore throat, or eruption on the skin, but I am, nevertheless, convinced that it is nothing but syphilitis. Why it selected the face in preference to the legs, or the skin instead of the lymphatic glands, hair, periosteum, or bones, no one can tell. You can judge positively from its appearance that this is not cancerous, as it has not the aspect of an epitheliomatous sore.

Now, what should be done here in the way of treatment? To treat it locally, only, would be the height of folly; we will, therefore, also give our constitutional remedies. The iodides, either of potassium, sodium, or ammonium, produce almost a specific effect in this disease, especially when combined, as I am in the habit of using them, with a small quantity of the bichloride of mercury. Nausea, produced either by too large doses or a too long continuance of them, must be avoided, as it would depress the patient. We commence, then, with a small dose, and will increase it judiciously.

℞.—Potassii iodid., gr. viij.; hydraag. chlorid. corros., gr. 1-10. M. Take either directly before, or an hour after, each meal. The diet must be light and nutritious, and skin must be kept clean, avoiding taking cold. As a local application to promote cicatrization—

℞.—Liq. hydrarg. nitrat., gr. x.; aquæ, f℥j. M. Use once a day, and have a cloth wet with—

℞.—Liq. plumbi subacetat., fʒj.; infus. ulmi, f℥viij., kept constantly on the part.

September 28.—Patient presented; much improved. Treatment continued.

October 10.—The ulcers have entirely healed under the treatment, thus verifying our diagnosis.—*Philadelphia Medical Times, November* 2, 1872.

*Coffee as a Vehicle for Quinine.*

M. Briquet (Bull. de Therap.) warns against the so common use of coffee as a vehicle for quinine. When a solution of the sulphate of quinine is put into tea or coffee, there occurs a decomposition of the salt, and the almost insoluble and insipid tannate of quinine is formed. This compound is almost inert, and the administration of quinine in this way, therefore, is one of the worst ever devised.—*Lyon Médicale, November* 10, 1872.

*Treatment of Scarlet Fever.*

The late Professor George T. Elliott, in a lecture on this disease, gave the following method of treatment: To bring the eruption out, if it has not already presented itself, order hot baths and blankets. Give nothing to eat at first in the eruptive stage, and only allow the simplest nourishment the first day. Patients experience great relief from baths, and the application of cold cream or mutton tallow over the whole body. Visit the patient twice a day. By pouring a pitcher full of cold water over the back of the neck, especially when the glands are enlarged, great comfort is experienced. As a gargle, make use of chlorate of potash or soda. Pieces of ice are good, in the mouth. Sprays, thrown in with Richardson's instrument, of lime water, solutions of alum and sulphate of zinc, are beneficial. As a palative to the throat, the vapor of slaked lime can be recommended. Strong beef-tea, with opium, may be thrown up the bowel. Begin to feed the patient from the second day of the eruption with animal essences. If the tonsils are enlarging and the pharynx exhibits much redness, with diphtheritic exudation, the physician has a right to say that things look bad. If the throat symptoms do not mitigate on the fourth or fifth day, then one feels that there is a good deal of danger. When the kidneys show hyperæmia, desquamation, or transitory albuminuria, then there is a twofold danger. Always examine the urine. When the patient has kidney disease, the treatment should be directed to the skin and bowels; when the latter are loaded and constipated, give powerful saline cathartics. Get Ronochetti's apparatus, to produce perspiration. To convalescing patients the use of iron is beneficial. The bisulphites have been recommended, but, from experience, they cannot be advocated. Belladonna is not always a prophylactic, although, on account of its innocence, and a feeling of satisfaction to the practitioner and family, it is well to administer it.—*New York Medical Record.*

*Subcutaneous Injection of Mercury in Syphilis.*

The injection of bichloride of mercury in the treatment of syphilis finds a warm advocate in Dr. Staub, of Strasbourg (Traitement de la Syphilis), who recommends the following formula: Bichloride of mercury, 1.25 grammes; chloride of ammonium, 1.25 grammes; chloride of sodium, 4.15 grammes; the white of one egg; distilled water, 250 grammes. One centigramme of this is injected daily beneath the skin, in two portions, one-half in the morning, the other in the evening. According to this somewhat enthusiastic writer, the bichloride, so administered, is an invaluable therapeutic agent, infallible in the treatment of secondary symptoms, prompt, complete, and devoid of inconvenience.—*Gaz. Hebdom.*

*The Relation of Obesity to Diseases of the Sexual Organs in Women.*

Dr. H. Kirsch, of Prague, (Centralbl. f. d. Med. Wis., No. 34), advances the idea that diseases of the sexual organs in women are not unfrequently dependent upon the presence of an abnormal amount of adipose tissue. Of two hundred and fifteen fleshy women whom he examined, two hundred and eight were found to suffer from some irregularity in the menstrual function. Of these two hundred and eight, one hundred and sixteen complained of scanty menstruation, (*menstruatio pauca*), one hundred and forty-six of leucorrhœa, and fifty-six of chronic metritis. Anteversion and anteflexion of the womb in thirty-nine of these cases; retroflexion in eleven; forty-eight were sterile, and forty-seven suffered from hysteria. Dr. Kirsch maintains that the majority of these various affections tend to improve, without further treatment, when once the obesity has been made to disappear. To attain this result, he recommends the adoption of a course of diet somewhat like that suggested by Banting, combined with a moderate use of the waters of Carlsbad and Marienbad.

*Oakum Dressings for Wounds.*

Oakum has long been employed in American hospitals as a dressing for wounds, more especially such as are attended with copious suppuration. Latterly, it has found its way into one of the hospitals of Königsberg, where, for the time being, its use has superseded that of all other dressings, and with satisfactory results. It is recommended by Dr. Heiberg, not as a disinfectant, but as possessing two important advantages over other applications:

1. The oakum may remain for a long time undisturbed. In some cases it has not been touched for eight days after the first application.
2. There is a marked diminution in the amount of pus secreted when oakum is used as a dressing.

*Sulphate of Quinia.*

Brother practitioners, you may remove much of the objectionable features to this valuable remedy by directing it taken in sweet cream, as this removes much of the bitter taste, and renders it much easier to be taken. It dissolves readily in cream, and children or adults take it much more readily. Try it, and your patients will thank you kindly.—*J. L. Hensley, M.D., in Eclectic Med. Journal.*

*Cancrum Oris.*

Mr. McGreevy writes that he has never found any application so effectual in this affection as hydrochloric acid. He applies it to the gangrenous spots with a feather or small brush.—*Practitioner.*

*Application of Electricity.*

An article of the Wiener Medical Presse quotes a communication made by Dr. Benedict to the Medical Society of Vienna, on the application of electricity to medicine. He declares that great results can be obtained from Fromholt's zinc and lead pile. He himself constructed a pile of zinc and carbon, which could be put in action by a solution of one gramme of bichromate of potash in fifteen grammes of sulphuric acid. He has made use of electricity in tumors and inflammatory swellings of the joints, by applying the current, not to the tumor itself, but to the nerves which ramify in it, or to that part of the spinal cord from which the nerves emanate; also for tumors containing liquid, such as hydrocele; for aneurisms, by introducing needles communicating with the positive pole; for lymphatic tumors, by introducing needles connected with the negative pole, and taking care to conduct only a moderate current, so as to avoid suppuration and alterations; and also for indolent buboes, neoplasms and cancers.—*British Medical Journal.*

*Night Sweats.*

Dr. J. O. Webster (Boston Medical and Surgical Journal) writes: In the number of the Journal for July 4th, you quote from Dr. Williams's work on consumption, "the medicine we have found to act as a specific on night sweats is the oxide of zinc, in doses of two or three grains in the form of a pill at night;" and you ask, "how closely will the experience of the American physician coincide with this statement?" While the surgeon to the National Military Asylum, Eastern Branch, 1868–'70, there were under my care many cases of consumption, mostly from two to five years' standing. Night sweat was a very common symptom, and for its relief I learned to rely entirely on the oxide of zinc, three grains in pill at night, combined with a little hyoscyamus. It seemed as nearly entitled to the name of specific as any medicine in the pharmacopœia.

*Disinfection.*

Pound the well-dried raw bean of coffee, and strew it over a moderately heated iron plate till the powder assumes a dark-brown tint; it will then remove almost any noxious effluvium.--*M. Weber, from Braithwaite.*

*Cholagogue.*

The following is advised as an excellent cholagogue to combat very obstinate hepatic troubles:

℞.—Fld. ext. taraxaci; elixir cinchonæ, ferri. et. strychniæ, aa. M. Dose, two drams three times daily before meals.

*A Mode of Operating for Radical Cure of Varicocele.*

Dr. H. B. Davison, San Francisco, (Pacific Medical and Surgical Journal), has adopted a new mode of operating for radical cure of varicocele, for which he claims three great advantages over any other means:

First, by perforating only one wall of the scrotum, less pain, less inflammation, and less risk of adhesion of the wounded sac and spermatic cord.

Second, by placing the patient in a recumbent posture when the operation is being performed, so that no blood may be inclosed in that portion of the vein cut off from the circulation, the resultant inflammation will be much less, and the testicle will not swell so much, and absorption will be accomplished in much less time.

Third, by removing the ligature before it cuts through the vein, the risk of phlebitis is lessened, and the patient is enabled to resume his ordinary duties much sooner.

Those who have been operated on have no return of the disease, and it would require a very close examination of the parts to discover that any operation had been performed. In one case the patient had been wearing a suspensory bandage for over twenty years, and the left testicle was much atrophied. It is now about sixteen months since the operation, and the testicle has regained its normal size, and the patient has a corresponding increase of sexual power. *The Clinic.*

*Uterine Hemorrhage.*

Dr. Lyman having alluded to the case of recurring uterine hemorrhage which he had reported at a previous meeting, Dr. Fifield said that he thought ferric alum, in the proportion of ℥ iij. of the salt to ℥ viii. of water, was the best astringent for injections. It gave rise to a less amount of "slag" than any other preparation of iron. In a case of cyst in the popliteal space which he had incised, there was a profuse hemorrhage which was readily controlled by the use of this styptic.—*Boston Medical and Surgical Journal.*

*Novel Method of Detecting Small Pox.*

An Italian physician can detect the presence of variola before the appearance of the eruption by an itching on his forehead and chin! He has verified the statement by a number of observations, and his assistants also confirm it by their own experience.—*Pacific Medical and Surgical Journal.*

*Castor Oil Emulsion.*

℞.—Ol. ricini, syr. fruct. aurant., aa ℥ i.; vitellus ovi, No. 1; aq. flor. aurant., ℥ s. M. ft. emulsio. This makes a very pleasant emulsion, which is readily taken by adults as well as children.

*Galvanic Treatment of Bed-Sores and Indolent Ulcers.*

Dr. Hammond, of New York, recommends for indolent ulcers and bed-sores the galvanic treatment, as first suggested by Crussel, of St. Petersburg. He says: "During the last six years I have employed it to a great extent in the treatment of bed-sores caused by diseases of the spinal cord, and with scarcely a failure; indeed, I may say, without any failure, except in two cases where deep sinuses had formed, which could not be reached by the apparatus. A thin silver plate—no thicker than a sheet of paper—is cut to the exact size and shape of the bed-sore; a zinc plate of about the same size is connected with the silver plate by fine silver or copper wire six or eight inches in length. The silver plate is then placed in immediate contact with the bed-sore, and the zinc plate on some part of the skin above, a piece of chamois-skin soaked in vinegar intervening. This must be kept moist, or there is little or no action of the battery. Within a few hours the effect is perceptible, and in a day or two the cure is complete in a great majority of cases. In a few instances a longer time is required. I have frequently seen bed-sores three or four inches in diameter, and half an inch deep, heal entirely over in forty-eight hours. Mr. Spencer Wells states that he has witnessed large ulcers covered with granulations within twenty-four hours, and completely filled up and cicatrizations begun in forty-eight hours. During his recent visit to this country, I informed him of my experience, and he reiterated his opinion that it was the best of all methods for treating ulcers oi indolent character and bed-sores."

*Blisters in Pneumonia.*

Dr. C. J. B. Williams, in speaking of pneumonia, says: "My experience has taught me to put great faith in large blisters, both in asthenic pneumonia and bronchitis, and I am confident that I have seen many lives saved by their means. Instead of being lowering, they give a salutary excitement to the circulation, and the copious serous discharge which proceeds from the skin tends to relieve the congested lung without wasting the blood, that is so necessary to sustain the functions. Small blisters tease as much as large ones, and are far inferior in the relief they afford.—*Am. Prac.*

*Treatment of Gonorrhœa.*

The fashion changes in this, as in other things. As a new fashion, I use, internally, in the acute stages: ℞.—Tinct. veratrum, gtts. xx.; gelseminum, ʒ j.; water, ℥ iv. Dose, a teaspoonful every two hours. As an injection, once or twice daily, as the acute stage is passing away: ℞.—Carbolic acid, grs. x.; tannic acid, grs. xv.; water, ℥ iv.—*Dr. J. M. Scudder, Eclectic Medical Journal.*

*Rapid Death from Stings of Bees.*

J. O. Sanders, M.D., reports the following: April 18th I was called to see a patient stung by bees. Mr. S., an intelligent man, gave the following account: Louis ——, a negro, aged about 45, climbed a tree where bees had swarmed on a limb, for the purpose of hiving them, carrying with him a saw. As soon as the limb commenced falling, the bees arose *en masse* and covered his head and face. He descended immediately, and, as soon as he reached the ground, commenced running as fast as he could; ran around three sides of a yard, some two hundred steps, passed through an open gate, and fell to the ground. Mr. S. ran to him with a bottle of spts. camphor, and succeeded in forcing him to take one swallow; the patient protesting at the time against assistance, declaring that he would certainly die. After two or three irregular respirations he expired. Mr. S. thinks it could not have been more than five minutes from the time he was attacked by the bees before he breathed his last. When I arrived, about an hour and a quarter after the accident, I could, on careful examination, find no signs of life. He was a vigorous, muscular man, and in perfect health, so far as I can learn. Was death due, in this case, to direct nervous shock, or to absorption of virus, or both?—*Medical News.*

*Changing the Direction of Wild Hairs.*

A new method of changing the direction of wild hairs, which turn in upon the ball of the eye, is recorded by Julian J. Chisolm, M.D., Professor of Eye and Ear Diseases, University of Maryland, in the August number of the Richmond and Louisville Medical Journal. The rationale of the operation is, that the hair drawn through the lid will, by constant traction in growth, change the position of its hair-bulb, and in this way correct the wild direction which it formerly took, to the serious injury of the patient.

Dr. Reynolds, of Louisville, (American Practitioner), has used the following injection in gonorrhœa with almost uniform success: ℞.—Copaibæ, ʒ x.; sodæ carbonatis, ʒ xx.; aquæ, ℥ xxx. *Fiat emulsio.* To one part of this emulsion are added three parts of water, and this is to be injected two or three times a day, through a catheter passed down the urethra to the stronghold of the disease. In chronic cases, equal parts of the emulsion and of water may be used.

Chronic Eczema of the hands, with fissures and cracks in all directions, is cured by M. Hardy, of Paris, by the use of India rubber cloth made to completely envelop the affected parts. Under the influence of this covering, the fissures become cicatrized, and the skin recovers its suppleness in forty-eight hours.

*Treatment of Constipation by Electro-Therapeutics.*

Dr. Cade (Lyon Médicale, No. 4, 1870) mentions the case of a lady of eighty, affected with habitual constipation which arose after dysentery, from which she had suffered at the age of twenty years. The author having tried various remedies for several months, and when the patient was in great danger of her life, he bethought himself that the sole method of causing peristaltic motion was electricity. Using the apparatus of Gaiffe, he applied the negative pole to the rectum, and the positive pole to the umbilicus. The induced current was made to act for twenty minutes, commencing with the least intense, and increasing up to No. 5 of the graduator. The sitting, although long and painful, was well supported, and the author had the satisfaction of seeing the patient relieved of her constipation by an abundant evacuation of solid fæces.

*Electricity in Angina Pectoris.*

Dr. Beard (Philadelphia Medical and Surgical Reporter, May 11) says that induced electricity was already spoken of by Duchenne and Trousseau in angina pectoris, when this presents itself as a true neuralgia, independent of the heart or great vessels. The author in a few sittings also obtained a rapid cure of an idiopathic angina pectoris, in a robust man of the age of 48. During the access, the positive pole was placed on the left breast, and a strong current was passed through the body. The attack disappeared immediately, and it was impossible for the patient to reproduce it even with violent exertion, whilst at first it arose when the slightest exertion was made.

*Abortive Treatment of Boils and Whitlow.*

Dr. Simon de Forges (Rev. de Thèrap.) advises the topical use of camphorated spirits as an abortifacient in boils and whitlow. In the former case the boil is to be rubbed eight or ten times by the finger dipped in the alcohol. He asserts that it is rare that, after this treatment, a boil goes on further towards suppuration. In cases of whitlow, he advises the patient to dip the finger for some ten minutes in camphorated spirits. This almost always gives great relief of the pain, and often cures the complaint.—*Medical News and Library, December,* 1872.

*Bromide of Calcium.*

We have published notices of this new salt several times during the year, and we now say that we have used it with marked advantage. To induce sleep, it is far superior to bromide of potassium. We give it in solution: ℞.—Bromide of calcium, ℥ ss.; simple syrup, ℥ ij. Dose, a teaspoonful.—*Dr. J. M. Scudder, Eclectic Medical Journal.*

*A Dangerous Paper.*

The green paper used to wrap about lozenges, sold in shops, railroad cars, and on the street corners, has long been suspected to contain arsenic, and with the view of ascertaining the facts by analysis, we recently purchased a roll of lozenges covered with this paper. A qualitative examination of the paper afforded all the characteristic reactions for arsenic and copper. The wrapper contained 20 square inches of paper. Of this, 16 were taken for quantitative analysis. The result of the examination showed that this portion contained 15.16 grams, or 2.34 grains of metallic arsenic. This is equivalent to 2.94 grains in the whole of the wrapper, a quantity sufficient to destroy life in an adult person. Children in all parts of the country are allowed to purchase the lozenges covered with this poisonous paper, and the rolls are often put into the hands of infants as a plaything. As everything goes into the mouth of young children, it is easy to see that no more dangerous substance can pass into a family than these packages of confectionery. It is quite probable that instances of poisoning have occurred from this cause, which have been of a serious or fatal character. There should be laws prohibiting the use of poisonous papers for any purpose.—*Boston Journal of Chemistry.*

*Aspiration and Iodine Injection in Catarrh of the Lachrymal Sac.*

M. Verneuil employs the following simple and ingenious method of treating catarrh of the lachrymal sac. He evacuates the contents of the sac by direct puncture with the needle and syringe of Pravaz, whereupon he injects the tincture of iodine. This procedure obviates the danger incurred by the least movement on the part of the patient when iodine was injected directly into the canaliculæ after the old plan. If the contents of the sac cannot be removed by aspiration, he forces in a few drops of iodine anyhow, and permits them to remain. This method of treatment is now in practice at the hands of many operators.—*Mouvement Médical, November* 10, 1872.

*Transplanting a Tooth.*

Dr. Gerhart, in the Dental Times, records a case in which a boy's tooth was knocked out, carried in the pocket twenty-four hours, and then replaced in its socket. At the end of four months this tooth looked as well as any of the boy's teeth, although it was necessary at the end of two months to devitalize it and pivot the root.—*West. Med Advocate.*

Pruritus Vulvæ, it is said, may be allayed, or even cured, by a wash of sulpho-carbolate of zinc of the strength of one-half drachm to the ounce of water.

*Aspiration in Hydrocephalus.*

Dr. Buttenwieser (Deutsch. Archiv. für Klin. Med., July 26, 1872) communicated a case of hydrocephalus in a boy of 26 weeks of age, treated by aspiration with syringe of Bergsen. The circumference of the head measured 54 ctm. The deformity of the head was very marked (*tête carée.*) The needle was entered to the left of the median line at a point of greatest prominence. The body of the syringe filled at once, and was emptied into a glass through the side tube. After the removal of about a quarter pound, both sides of the head had collapsed considerably, and the needle was withdrawn. The whole operation was performed without accident. The child cried throughout, and became pale towards its close.

Chemical examination of the fluid showed water 98.34, albumen 0.33, organic matter 0.41, ash 0.82. The organic matter consisted of fat, cholesterine, and traces of urea. The ash was chiefly composed of common salt and phosphate of soda.

The wound was closed with plaster. For a few days there was no change in the child's condition, but the fluid had reaccumulated in 24 hours to its former amount. Compression with plaster bandages was now undertaken. The child became worse, the fluid continued to accumulate with all the symptoms usual to compression, and death occurred in 11 days after the operation.—*Medical Times.*

*Hooping-Cough.*

To a child five years of age give the thirty-second part of a grain of morphia, with three grains of bromide of potassium, in solution, every two hours; letting the mother be instructed to suspend the medicine for four hours at any time, if unusual drowsiness comes on.—*Braithwaite's Retrospect.*

*Incontinence of Urine in Children.*

Mr. Holmes Coote recommends for this intractable affection the administration of creosote in one minim doses, three times daily, combined with assafœtida and rhubarb pill, of each two grains.—*New York Medical Record.*

A USEFUL form of disinfectant has been recently brought into use in England, namely, sawdust soaked in a saturated solution of carbolic acid. It is convenient for many purposes, cheap, easily prepared, and not liable to be swollowed accidentally, as ordinary liquid disinfectants are.

INK spots may be removed from colored fabrics by a concentrated solution of sodium pyrophosphate, which dissolves the ink slowly without affecting the color of the fabric.—*American Journal of Pharmacy.*

*To Prevent Abrasion of the Cheek from Spiral Springs.*

When it is necessary to use spiral springs for the first time, much discomfort is often suffered by the patient on account of the chafing produced by them. This may be, in a great measure, prevented or relieved by one or other of the following plans: Either make a solution in chloroform of pink rubber and paint over the surface of the springs before putting them on the set of teeth, or else obtain some of the very fine and thin elastic rubber tubing which is now made; and, having first passed a broach up the center of the spring to prevent crippling, draw this tubing over, so that the whole of it is covered except those portions that play against the teeth. In this way these necessary evils may be made tolerable to sensitive mouths by protecting the mucous membrane from their metallic feeling and irritation.—*O. C., in Monthly Review of Dental Surgery. Dental Cosmos.*

*Preserving Vaccine Virus.*

Dr. D. B. Hillis (American Journal of Dental Science, from the Pharmacist) recommends the preservation of vaccine virus between layers of dental red rubber, and says that, for several years past, he has used no new matter, but has, for the sake of experiment, drawn from the stock originally put up as described. He has always found it effective without paying any regard to temperature. A short time ago he successfully vaccinated a patient from a crust which had been laying about his office twenty-seven months.—*Philadelphia Medical Times*

*Fluid to Restore Putrid Tissues.*

Dr. B. W. Richardson gives the following formula for a restorative fluid, which he says will render soft, putrid tissues firm and inoffensive, so that they may be readily dissected: ℞.—Iodine, ʒ i.; methylated ether, sp. gr. ·720, f ℥ x.; absolute alcohol, f ℥ x.; strong sulphuric acid, f ʒ iv. Dissolve the iodine in the ether and alcohol mixed together, then slowly drop in the sulphuric acid.—*New Remedies.*

*Carious Teeth.*

M. Magitot recommends the following formula: Chloroform 5, laudanum 2, and tinct. benzoin 10 parts. Cotton wool dipped in this is to be inserted in the cavity, and renewed until insensibility of the part is produced, when the cavity may be definitely obdurated.—*Rev. Med.*

*Stings of Bees and Wasps.*

Inject into the puncture a solution of carbolic acid. The pain will instantly cease.

*Herpes Zoster Treated by Electricity.*

Dr. Picot (Gaz. de Hôp., 1870, No. 96) speaks of a lady, æt. 45, who for a week had suffered from severe pains in the left side of the chest, which deprived her of sleep; and then was followed by an eruption of little pustules, which dried up in fifteen days, but returned in five weeks. Dr. Picot, of Tours, made the diagnosis of intercostal neuralgia, and thought of applying a continuous current obtained from an apparatus of Remak. The positive pole was run along the spines of the cervical vertebræ, and the negative pole applied along the course of the affected nerves for ten minutes each nerve. This effected a cure, and the eruption and pain disappeared.—*Doctor.*

*Cancer.*

The most recent views of the nature of cancer are, that it is not a constitutional disease at all at first, but purely local, and that, by subsequent absorption of its elements, the system becomes affected. It may be said to be hereditary, in the same way as a tendency to any other affection (as warts) is hereditary. The use of caustics after the removal of the mass of the tumor by the knife is coming more and more into favor. Chloride of zinc is especially suitable. *Braithwaite's Retrospect.*

*Carbolic Acid in Gonorrhœa.*

Mr. John Ashmead states that he has been using, for the last five months, carbolic acid in combination with glycerin and tannin, as an injection in cases of gonorrhœa, and has found it quite as efficacious as Mr. Wood described in a former contribution. The formula employed consists of eight grains of carbolic acid, eight grains of tannic acid, half an ounce of glycerin, and water to one ounce. It appears to act as an antiseptic, arrests the discharge, and shortens the course of the disease.—*Medical Examiner.*

*Blood in the Urine.*

Blood is very rarely seen in simple cystitis, but it is as common in vesical calculus as hæmoptysis is in phthisis, and is especially common after some exertion, as driving or riding. Blood from this cause is rather florid in tint, whilst blood passed from the kidney remains long in the bladder, and from contact with urine is brown in color. Hemorrhage may also occur in hypertrophy of the prostate.—*Braithwaite's Retrospect.*

*Whitlow—Carbolic Acid.*

After freely slitting up the parts and giving vent to the matter and sanious discharge, use a solution of carbolic acid freely. The wound will rapidly assume a healthy character.

*Bromide of Iron.*

This remedy is advocated by Dr. N. H. Norris, of Beloit, Wisconsin, (Northwestern Medical and Surgical Journal), as nearly a specific in involuntary seminal emissions and spermatorrhœa. He has administered it three times daily, an hour before or after meals, in doses of from three to five grains, rubbed up in a little syrup. Professor Namais (Practitioner) states that this remedy corrects defective formation of blood, quiets nervous excitation, and produces the combined effect of iron and the bromides. He regards bromide of iron as being in many instances a therapeutic agent of superior value in epilepsy even to the bromide of potassium.—*N. Y. Medical Record.*

*Injection of Alcohol into Cysts.*

At the Paris Surgical Society, M. Monod lately related some cases in which, after withdrawal of a portion of the fluid from cysts, he injected a little alcohol. The first case was that of a large cystic goitre. One injection greatly diminished the size, and the second, made a fortnight later, completed a cure in a month from the first operation. He was more successful still in hydrocele. The process, he says, does not destroy the tunica vaginalis, but restores it to a normal condition. The alcohol effects a change that enables absorption no longer to be overbalanced by secretion. M. Monod thinks this method of treatment applicable to ovarian and other cysts.

*Chronic Gastritis.*

We can see with the eye the effect of morphia in some cases of acute sclerotitis and iritis; why should not the same "antiphlogistic" power be exercised in other painful inflammatory affections? Morphia is a remedy of some value in chronic gastritis. Let a twelfth of a grain be given twice on the first day, three times on the second, and so on until the patient consumes from one to one and a half grains in the twenty-four hours.—*Drs. Barclay and Stokes, Med. Chir. Review, Jan., p.* 218.

*Treatment of Ganglion.*

The treatment which Dr. Fiddes (Aberdeen Royal Infirmary) adopts with regard to ganglion on the wrist or hand is puncture with a grooved needle, pressing the fluid out of the sac, and afterward applying constant pressure. Dr. Fiddes never saw any outward application, such as iodine, paint, etc., do good.

To quiet the aching of a carious tooth, introduce into the cavity a small quantity of the solid hydrate of chloral.—*American Journal of Dental Science.*

*Knot in the Cord.*

Dr. N. W. Webber, of Detroit, has met with a case in which a simple knot was found to be tied in the funis. This could only have been done while the fœtus was very small, since the end of the cord must have been passed through the loop. The activity of the very young fœtus is doubtless much greater than is commonly supposed. The women think that the life of the child begins about the fourth month when it is first *felt*, but this is undoubtedly a mistake. The early movements are not felt probably only because the child is small and its limbs do not strike the uterine walls. The amniotic fluid is abundant and protects the uterus from these blows.

W. Tyler Smith advanced the idea many years ago, that *none* of the spasmodic sensations of the mother supposed to be due to the movements of the fœtus were so caused. He attributed all of these sensations to uterine contractions. His hypothesis, however, did not meet with the endorsement of the profession, and he has now abandoned it himself.

In Dr. Webber's case, the knot was tied loosely, so that the circulation was not sufficiently impeded to destroy life. The child looked a little blue at first, however, and its prospects were dubious. *Western Medical Advance.*

*Dust in the Air-Passages.*

Dr. W. H. Bennett, in the New York Medical Record, calls attention to the danger of acquiring pulmonary disease in this way. In the tobacco factories of Detroit we have many cases of phthisis, caused by the dust of tobacco, licorice and other substances which are subjected to grinding.—*Western Medical Advance.*

*Animalculæ in Buttermilk.*

Several persons were recently made very sick at Galt, Ontario, by drinking buttermilk, which tasted sweet and was fresh from the churn. How long previously the milk had been taken from the cow, we are not informed. When examined microscopically it was found to contain animalculæ in large quantities.—*Canada Lancet.*

*Syphilis.*

Dr. Kearney in the Cincinnati Lancet discusses the present views held with regard to the chancroid and chancre. He says that the prevailing tendency—the tidal wave—is now favoring the old idea of there being one disease. In fact, even a gonorrhœa in woman may cause syphilis in the man.

Dr. W. F. Jenks, of Philadelphia, has used nitrite of amyl in *puerperal convulsions* with success. He allowed the patient to inhale the medicine from a handkerchief in the usual manner, some five drops only having been used.

*Cundurango is not Dead Yet.*

This drug is again coming to the surface. Professor Andrews, of Chicago, has received many letters from physicians practicing in Guayaquil, Quito and Laja, assuring him that cundurango is by no means a valueless drug. It is regarded as of some value for cases of cancer, but as a cure for *syphilis*, it is unrivaled. The natives unite with the cundurango and extract from a bark called *oriental*, which greatly increases its power. This oriental extract is an emetico-cathartic, and has an admirable effect in diseases of the liver, stomach and intestines. It will be remembered that when cundurango first appeared, we were informed that a woman in South America tried to poison her husband with it, but to her great disappointment she cured him instead, and that the husband's disease was *cancer*. We are now assured that this first patient had not cancer, but *syphilis*. Further details will be found in the Chicago Pharmacist.—*Western Medical Advance.*

*Amputation of the Cervix Uteri by Means of the Galvanic Cautery.*

Dr. James T. Whittaker, in the Clinic, reports such an operation which he performed upon a patient suffering from sterility and various menstrual irregularities. There was "cervical elongation to the extent of an inch; anteflexion with pressure upon the bladder and impermeability to the entrance of even the finest sound." The patient recovered well from the immediate effects of the operation, but sufficient time has not elapsed to decide whether a permanent benefit will be secured.

*Carbolic Acid in Pruritus.*

In prurigo and pruritus, says Dr. Pintschovius, in the Allgemeine Medicinische Central Zeitung, I have successfully tried carbolic acid externally. I prescribe a solution containing 2½ per cent. of carbolic acid, and of this direct a tablespoonful to be mixed with a teacupful of rain-water. Every morning and evening the diseased skin is thoroughly sponged with this. I treated thirty patients in this way, and every one has recovered in from three to eight days' time.—*Medical and Surgical Reporter.*

*Arsenic in Red Paper Hangings.*

Dr. N. Hallwachs has discovered arsenic, in considerable quantities, in gray and red paper hangings, so that, not only green papers, but those of other colors, must be looked upon with suspicion.

*Constipation.*

Take of podophyllin 6 grains; extract of nux vomica 7½ grains; extract of belladonna 4 grains; mix, and form into 12 pills. Dose, one, two, or three pills a day, according to their action.

*Effects of Color on Health.*

A correspondent of The Builder states that he had occasion to examine rooms occupied by young ladies for manufacturing purposes, and he has observed that while the workers in one room would be very cheerful and healthy, the occupants of a similar room, who were employed on the same kind of business, were all inclined to be melancholy, and complained of a pain in the forehead and eyes, and were often ill and unable to work. The only difference he could discover in the rooms was that the one occupied by the healthy workers was wholly whitewashed, and that occupied by the melancholy workers was colored with yellow ochre. As soon as the difference struck him he had the yellow ochre washed off the walls and then whitened. At once an improvement took place in the health and spirits of the occupants.—*Med. and Surg. Reporter.*

*Itch.*

The best remedy is *Peruvian Balsam*—forty drops five times a day rubbed on carefully when the skin is dry. This quantity is sufficient for the whole body of a child. Next to Balsam of Peru, *carbolic acid* is recommended. It may be dissolved in glycerin or linseed oil, ten grains to the ounce. The preliminary cleansing with warm water and soap is also recommended.—Prof. Rothmund, of Berlin—*Canadian Pharmaoeutical Journal.*

*Ipecacuanha in Epistaxis.*

Dr. John Shrady, of Harlem, New York, has successfully exhibited ipecacuanha in several severe cases of epistaxis, especially in the form associated with chronic alcoholism. In one instance, where from a previous experience, plugging the posterior nares was strongly objected to, he used vinum ipecacuanhæ in teaspoonful doses until free emesis was produced with the result of arresting the hemorrhage in fifteen minutes.

*Bromide of Potassium in Leucorrhœa.*

Dr. A. H. Kinnear, Metamora, Illinois, (Chicago Medical Journal), has given, with success, in twelve marked cases of vaginal or uterine leucorrhœa, none of which were of less than six month's standing, bromide of potassium. The majority of the cases yielded to the treatment in four weeks. He has observed two effects from the use of this drug: the alterative and nervosedative.

*Camphor in Erysipelas.*

Dr. Delpech recommends an ethereal solution of camphor, composed of equal weights of both, a few drops of which are from time to time put upon the erysipelatious surface. In most cases a rapid cure follows.—*American Journal of Pharmacy.*

*New Method of Making Beef-Tea.*

Dr. H. C. Wood says: In order to meet the daily felt want of concentrated fluid meat food, a want not supplied by beef essence as ordinarily made, I have invented the following process, and found in practice that it works well. Take a thin rump steak of beef, lay it upon a board, and with a case-knife scrape it. In this way a red pulp will be obtained, which contains pretty much everything in the steak, except the fibrous tissue.

Mix this red pulp thoroughly with three times its bulk of cold water, stirring until the pulp is completely diffused. Put the whole upon a moderate fire, and allow it to come slowly to a boil, stirring all the time to prevent the "caking" of the pulp. In using this do not allow the patient to strain it, but stir the settlings thoroughly into the fluid. One to three fluid ounces of this may be given at a time.—*New Remedies.*

*On the use of the Bowl during Delivery.*

Dr. J. Mathews Dancan, in a paper read before the Obsteterical Society of Edinburgh, recommends the use of an ordinary wash bowl in the place of cloths for the purpose of catching the discharges which come from the vulva during labor. It has the advantage of cleanliness, is inexpensive, guards against cold by the removal of the liquor amnii, blood, meconium, and clears the air of noxious germs and decomposing animal matter; and enables the attendant to estimate the quantity and quality of the discharges.—*Boston Medical and Surgical Journal.*

*Sunstroke.*

Dr. C. E. Wright contributes an interesting article on sunstroke to the Indiana Journal of Medicine. As there have been many cases lately, it is important to know that no new methods of treatment have been generally adopted. The indications are to keep the patient cool and gently stimulate him until the pulse becomes strong. In cases of an apoplectic character, however, with a full pulse at the outset, antiphlogistic measures alone should be employed.

Dr. Bell, in the Glasgow Medical Journal, calls attention to the superiority of the elastic handage over the elastic stocking. It is cheaper, easier to apply or remove, and renders it possible to exercise pressure or support on any particular part.

*Prescription for Softening of Bones in Children.*

℞.—Calcia Phosphatis, ʒ ij.; calcis carbonitis, ʒ i.; sacch. lactis, ʒ iij.; M. S.—10 or 20 grains two or three times a day in sweetened milk.

*Tincture of Calabar Bean.*

Take of calabar been in No. 50 powder, sixteen troy ounces; acetic acid, one half fluid ounce; strong alcohol and water, of each sufficient; mix the acid with three pints of water; pour this upon the powder and set aside the mixture for a few days, in a warm place, that the legumin may undergo putrefaction; then add three pints of strong alcohol, and macerate a few days longer; after this pour part of the mixture on a muslin strainer, press the liquor out and pack the residue firmly into a cylindrical glass percolator having a broad base; pour upon this the rest of the mixture, together with the strained liquid, and then diluted alcohol until eight pints of percolate have passed.—*R. Rother, in Chicago Pharmacist.*

*Antidotes to Carbolic Acid.*

The Philadelphia Reporter gives us the following in regard to carbolic acid: "Dr. T. Husemann ("memorabilien," 1872) says that oils can be used as antidotes in poisoning by carbolic acid or creosote. Curara is no antidote. Carbolate of potash and the metals are as poisonous as carbolic acid itself. Chalk is not altogether useless as an antidote, but is not so useful as the saccharine carbonate of calcium. The greatest hope as an antidote is to bring about the oxydization of the carbolic acid. A stomach pump, if at hand, is the best remedy in poisoning by carbolic acid; emetics do more harm than good."

*Chloride of Potassium in Epilepsy.*

Dr. Lander (Echo Mèd. et Pharm. Belge.) advocates this salt as better than bromide of potassium in epelepsy. He finds it is more active, costs five-sixths less, and has not the inconvenience of the secondary effects of bromide of potassium. He begins with small doses, and has continued the use of the drug for several months without any bad consequences, in daily doses of from 3 grammes 50, to 5 grammes 50 (1 to 2 drachms). Moreover, Dr. Lander thinks that the bromide is converted into a chloride in the stomach, so he suggests the immediate use of the chloride.

*Amaurosis from Smoking.*

There is undoubtedly a form of amaurosis produced by tobacco smoking. Excessive smoking will not in some people have any such effect, for this deplorable result is due to some extent to an idiosyncrasy. It fortunately happens that there is sufficient warning of the failure of sight, and if the habit is resolutely broken off, complete recovery results.—*Braithwaite's Retrospect.*

THE Eclectic Medical Journal calls attention to the value of nitric acid in the nutrition of nerves.

